# Role of Adjuvant Radiotherapy in Non-Small Cell Lung Cancer—A Review

**DOI:** 10.3390/cancers14071617

**Published:** 2022-03-23

**Authors:** Krisztian Süveg, Ludwig Plasswilm, Thomas Iseli, Pawel Leskow, Galina Farina Fischer, Paul Martin Putora

**Affiliations:** 1 Department of Radiation Oncology, Kantonsspital St. Gallen, 9007 St. Gallen, Switzerland; ludwig.plasswilm@kssg.ch (L.P.); thomas.iseli@kssg.ch (T.I.); paulmartin.putora@kssg.ch (P.M.P.); 2 Department of Radiation Oncology, University of Bern, 3010 Bern, Switzerland; 3 Department of Thoracic Surgery, Kantonsspital St. Gallen, 9007 St. Gallen, Switzerland; pawel.leskow@kssg.ch; 4 Department of Radiation Oncology, Kantonsspital Winterthur, 8401 Winterthur, Switzerland; galina.fischer@ksw.ch

**Keywords:** NSCLC, PORT, radiation therapy, resection, risk factors

## Abstract

**Simple Summary:**

The role of postoperative radiotherapy (PORT) in completely resected non-small cell lung cancer (NSCLC) with ipsilateral mediastinal lymph node involvement (pN2) is controversial. The aim of our review was to study the literature relating to PORT for completely resected NSCLC patients with pN2 involvement. The Lung ART and PORT-C trials indicate better locoregional control with PORT, but this has not yet translated into survival benefits. Given the conflicting results, guidelines do not recommend the use of PORT routinely. Future research should focus on identifying subgroups of patients who might benefit from PORT.

**Abstract:**

Background: For patients with completely resected non-small cell lung cancer (NSCLC) with ipsilateral mediastinal lymph node involvement (pN2), the administration of adjuvant chemotherapy is the standard of care. The role of postoperative radiation therapy (PORT) is controversial. Methods: We describe the current literature focusing on the role of PORT in completely resected NSCLC patients with pN2 involvement and reflect on its role in current guidelines. Results: Based on the results of the recent Lung ART and PORT-C trials, the authors conclude that PORT cannot be generally recommended for all resected pN2 NSCLC patients. A substantial decrease in the locoregional relapse rate without translating into a survival benefit suggests that some patients with risk factors might benefit from PORT. This must be balanced against the risk of cardiopulmonary toxicity with potentially associated mortality. Lung ART has already changed the decision making for the use of PORT in daily practice for many European lung cancer experts, with lower rates of recommendations for PORT overall. Conclusions: PORT is still used, albeit decreasingly, for completely resected NSCLC with pN2 involvement. High-level evidence for its routine use is lacking. Further analyses are required to identify patients who would potentially benefit from PORT.

## 1. Introduction

Multimodal therapy is the standard of care for patients with locally advanced (LA) non-small cell lung cancer (NSCLC) (stage IIIA-C) [1,2,3,4]. Patients with stage III N2 NSCLC with primarily unresectable disease (IIIA4, IIIB) receive concurrent or sequential chemoradiotherapy (CRT) followed by immunotherapy in some cases [5]. Alternatively, a surgical approach (bi- or trimodal) can be offered to patients with potentially resectable stage III NSCLC (IIIA3) [6,7]. Two meta-analyses confirmed that definitive CRT and a bimodal treatment including surgery and chemotherapy, were comparable options for treating N2 disease [6,7]. Feasible treatment options should be discussed by a multidisciplinary team. In a setting with multiple treatment options, patient preference should play a prominent role in the decision making [8].

After surgical resection, a large proportion of patients develop local recurrence (LR) and distant metastasis [9]. The poor prognosis after surgery has led to the implementation of perioperative treatments. The benefits of chemotherapy in patients with completely resected NSCLC have been demonstrated in many phase 3 studies and a meta-analysis, with an absolute survival benefit of 5% at 5 years [10]. Neoadjuvant or adjuvant chemotherapy is recommended for patients with stage IIB to IIIA and can be considered for patients with stage IIA with a resected primary tumor of >4 cm [1,2,11]. Nodal positive patients have a significant risk of locoregional recurrence even after R0 resection and neoadjuvant or adjuvant chemotherapy [9,12].

First data show that neoadjuvant immune checkpoint inhibitors (ICIs) (e.g., nivolumab) plus chemotherapy increase the rate of pathological complete response compared with chemotherapy alone (Checkmate 816 [13]). Likewise, in the same setting, adjuvant ICIs with anti-PD-L1 agents (e.g., atezolizumab or pembrolizumab) lead to increased disease-free survival (DFS) versus best supportive care for patients with PD-L1-positive tumors (IMpower010 [14], PEARLS [15]). The role of (neo)adjuvant immunotherapy in stage IB–IIIA NSCLC is rapidly developing and several large randomized phase 3 clinical trials are underway [11,13,15]. Osimertinib is indicated for the adjuvant treatment after complete resection with stage IB–IIIA EGFR-mutated NSCLC patients [11,16].

Postoperative radiation therapy (PORT) is often recommended in stage III N2 NSCLC patients, but its role has historically been controversial.

This review focuses on the role of PORT in the multimodal treatment of LA-NSCLC. We discuss how recent results may impact our current treatment approach [5].

## 2. PORT after Complete Resection

The International Association for the Study of Lung Cancer (IASLC) staging committee defined complete resection as follows: microscopically free resection margins (R0), systematic nodal dissection or lobe-specific systematic nodal dissection, lack of extracapsular nodal extension (ENE) and negativity for tumor infestation at the highest mediastinal node removed [17,18,19].

Between 2017 and 2020, three different studies confirmed the prognostic value of the IASLC definition [17,18,19].

### 2.1. pN0

A randomized trial from 1980 with 175 completely resected pN0 NSCLC patients showed that PORT was clearly detrimental [20]. The same team highlighted a decade later the potential benefit of modern treatment techniques, although the oncological results were not superior to those of the control group [21]. A more recent randomized trial using linear accelerators was similarly performed for 104 completely resected stage I patients. The 5-year overall survival (OS) showed a positive trend in the treated group (PORT group: 67% vs. non-PORT group: 58%, *p* = 0.048) [22].

PORT meta-analyses have shown a detrimental effect of PORT for completely resected NSCLC with pN0 and pN1 disease [23,24,25,26].

The ANITA trial was a randomized trial of adjuvant chemotherapy vs. observation in completely resected stage IB to IIIA NSCLC patients, revealing a significant 5-year OS benefit of 8.6% for the chemotherapy group [27]. The post hoc analysis of the ANITA trial study also showed a negative effect of PORT for pN0-1 patients [26].

Based on these data, PORT is not recommended for completely resected pN0 and pN1 NSCLC patients.

### 2.2. pN+

PORT was evaluated in several trials performed in the 1980s–1990s. An older retrospective study from the Mayo clinic, including 224 pN2 patients resected between 1987 and 1993, found that PORT may improve local control (4-year LR 60% in the non-PORT group vs. 17% in the PORT group, *p* < 0.0001) and survival (4-year OS 22% in the non-PORT group vs. 43% in the PORT group, *p* = 0.005) [28]. The largest randomized trial, including 728 stage I-III NSCLC patients, demonstrated that PORT had a detrimental effect on survival without a significant effect on LR [29].

The PORT meta-analyses have shown a deleterious effect of PORT for completely resected stage I–III NSCLC [23,24,25]. In patients with pN2 disease, PORT did not improve survival but did reduce the risk of local recurrence by 24%. Due to these negative results in the whole patient cohort, fewer patients were treated with PORT, even those in pN2 stage. The meta-analyses were criticized due to the use of obsolete two-dimensional (2D) radiation techniques (cobalt-60, not CT planned), suboptimal and outdated radiotherapy volumes and fractionation schemes often using daily fractions of >2 Gy, which might have potentially led to additional toxicities [30,31,32]. A comparison of RT plans used in older vs. current trials showed poor target coverage and excessive heart and lung doses leading to high toxicity with older techniques [33]. The included older studies were performed without ^18^FDG PET-CT staging or brain MRI imaging. Finally, chemotherapy was not used [34,35].

Since the PORT meta-analysis in 1998, several retrospective population-based cohort studies, database analyses and meta-analyses have found significantly improved OS for PORT in patients with completely resected NSCLC with pN2 involvement (Table 1).

A larger cohort study of the Surveillance, Epidemiology, and End Results (SEER) database with 7465 resected NSCLC patients suggested that PORT in pN2 patients was associated with an increase in cancer-specific survival and 5-year OS [36].

In the randomized ANITA trial, PORT was recommended for pN+ disease but was not randomized or mandatory [27]. A retrospective post hoc subgroup analysis demonstrated that PORT led to improved OS in patients with resected pN2 NSCLC both in the chemotherapy arm and observation arm [26] (Table 1). This trial was initiated in the era of adjuvant chemotherapy.

The meta-analysis of Billiet et al. [37] (2387 patients) based on phase 3 randomized controlled trials (RCTs), compared the effect of PORT in patients treated on linear accelerators or cobalt machines. PORT significantly decreased LR from 30% to 10% for pN2 patients independently of the radiotherapy machine used. Better OS was only achieved when PORT was delivered with a linear accelerator. Most of the patients in the studies were treated with 2D radiotherapy, which is no longer used in daily practice [37].

Another meta-analysis of 16 trials with 3278 patients indicated that PORT delivered with modern techniques, significantly improved locoregional recurrence-free survival, DFS and OS in patients with stage III N2 NSCLC [38].

A recent meta-analysis by Zhang et al. [12] summarized all studies (three RCTs [39,40,41] with 237 patients and eight retrospective studies with 7748 patients) regarding the effect of PORT on OS and DFS in stage III pN2 NSCLC. The results revealed that the use of PORT tends to prolong OS and significantly improves DFS. The effect of PORT on OS did not differ significantly between RCTs or retrospective studies [12].

A retrospective single center study by Wei et al. [42] with 183 patients from the Hunan Cancer Hospital in China demonstrated a significant improvement in LR-free survival and OS in the postoperative chemoradiotherapy (CRT) versus the postoperative chemotherapy group in stage III pN2 NSCLC, especially in the multiple-station pN2 patients and patients with single-station pN2 combined with multiple-station pN1.

A recently published retrospective study by Wang et al. [43] with 142 pN2 NSCLC patients found that PORT can increase the OS of patients (5-year OS 32% vs. 27%) as well as the local control rate of tumors.

Retrospective studies, large database analyses and meta-analyses on PORT for pN2 completely resected NSCLC patients from the last 20 years (Table 1) show controversial results. Our understanding of the use of PORT in the modern setting with a better selection of patients with ^18^FDG PET-CT and brain MRIs, improved radiotherapy and thoracic surgery techniques is limited. In the daily routine, PORT was mostly based on the risk of locoregional relapse for each individual patient. Two phase 3 randomized trials, Lung ART and PORT-C [44,45], were published in 2021 and brought more insight to this debate.

The Lung ART trial compared mediastinal PORT with no PORT in a superiority design in patients with completely resected NSCLC with pN2 involvement with modern surgery and 3D conformal radiotherapy [45]. In total, 91% of the patients were staged preoperatively with ^18^FDG PET-CT in this trial, and 96% of the patients received neoadjuvant or adjuvant chemotherapy. Five hundred and one patients were enrolled and randomized after resection or adjuvant chemotherapy: 252 in the PORT group and 249 in the control arm (no PORT). Only patients who had undergone a complete resection were included. The advisory surgical quality assurance committee reclassified resections based on the IASLC definition into R0, an uncertain resection, or R1 (because of ENE). The trial did not meet the primary endpoint of significantly improved 3-year DFS (47.1% in the PORT arm vs. 43.8% in the control arm, *p* = 0.18). Three-year OS was 66.5% in the PORT arm vs. 68.5% in the control arm. Twenty-five percent of the PORT patients and 46% of the non-PORT patients had mediastinal relapse at 3 years; this is a reduction of approximately 50% in the risk of locoregional relapse using PORT compared with the control group. Most patients died of recurrence: 85% in the non-PORT group and 69% in the PORT group. Cardiopulmonary toxicity and related death was significantly higher in the PORT group (16 patients, 16%) than in the control group (2 patients, 2%). The Lung ART authors concluded that 3D conformal PORT cannot generally be recommended for all stage III pN2 patients after a (considered) complete resection, although PORT could significantly reduce the risk for mediastinal relapse. In the PORT group there were more toxicities (especially cardiopulmonary) without a statistically significant effect in terms of OS. Further analyses are needed to determine if certain patients could benefit from PORT. It may be noted that the initial target accrual was 700 patients, yet the trial closed with 501 patients. However, it is unlikely that the results would have been significantly different with the initially planned accrual [46].

Two other RCTs comparing PORT versus no PORT were published between 2014 and 2020. One study closed early because of poor accrual [40]. The study of Sun et al. [41] recruited only patients with unsuspected N2 disease.

The Chinese RCT (PORT-C, *n* = 394) showed no significant difference in 3-year OS (78.3% in the PORT arm vs. 82.8% in the non-PORT arm) for completely resected NSCLC patients with pN2 involvement. The 3-year LR-only rate was significantly lower in the PORT arm (9.5% vs. 18.3%). The authors stated that the low toxic effects in this trial were due to the use of a modern RT technique (89% IMRT) and to the markedly tighter dose restrictions to the organs at risk. Nevertheless, IMRT did not improve OS, with similar death rates in the PORT and non-PORT arms [44].

However, further analysis is needed to identify cohorts that may potentially benefit from PORT without increasing the cardiopulmonary toxicities and potentially related deaths.

Based on all these data, guidelines do not recommend PORT routinely. The NCCN guidelines recommend PORT alone or with chemotherapy for selected pN2 patients only [2]. The updated ESMO Clinical Practice Guidelines see no benefit of PORT for patients with completely resected stage III N2 NSCLC and recommend PORT only in the setting of residual microscopic or macroscopic disease [11]. The recently published ASCO guidelines do not recommend PORT for patients with completely resected NSCLC with mediastinal N2 involvement without extracapsular extension who have received neoadjuvant or adjuvant platinum-based chemotherapy [47].

### 2.3. Importance of Surgery and Preoperative Staging from the Perspective of Modern PORT

In the last decade there has been major progress in terms of preoperative staging. Patients undergoing multimodality treatments are better selected based on modern staging with ^18^FDG PET-CT and brain MRI. ^18^FDG PET-CT is highly sensitive and specific in detecting mediastinal nodal and extracranial metastases. In total, 91% of the patients in the Lung ART trial had undergone ^18^FDG PET-CT scans. The ESMO guidelines recommend a locoregional lymph node staging with ^18^FDG PET-CT and, in the case of PET-positive lymph nodes or central or >3 cm tumors, an invasive staging with EBUS/EUS (endoscopic bronchial ultrasound) or VAM (video-assisted mediastinoscopy) [11].

The European Society of Thoracic Surgeons (ESTS) defined adequate intraoperative lymph node staging as: a systematic nodal examination including at least three intrapulmonary and hilar nodes and at least three mediastinal nodal stations depending on the location of the primary tumor [48]. Handa et al. [49] advocate for lobe-specific mediastinal lymph node dissection. One of the most important findings of the study is that nearly 6% of patients in the lobe-specific dissection group might have had their metastatic lymph nodes missed by not sampling stations outside of the lobe-specific stations.

### 2.4. Sequence of Postoperative Treatment

Sequential chemotherapy and PORT were associated with superior survival compared with concomitant postoperative CRT in two National Cancer Database (NCDB) registry analyses for pN2 NSCLC patients [50,51].

In the randomized trial of the Eastern Cooperative Oncology Group, PORT (50.4 Gy) alone was compared to postoperative CRT (with cisplatin and etoposide). The 3-year OS rates were similar in both groups [52].

In the RTOG 9705 trial, 86 patients with completely resected NSCLC underwent PORT (50.4 Gy) and chemotherapy (paclitaxel plus carboplatin) concomitantly. This trial evaluated the efficacy of combining chemotherapy and radiation therapy postoperatively. The trial suggested improved 3-year progression-free and OS rates (50% and 61%, respectively), compared with previously reported trials [53].

ESMO guidelines recommend administering chemotherapy first when both postoperative chemotherapy and PORT have been used [1].

### 2.5. Risk Factors

Stage III pN2 NSCLC is a very heterogeneous group with different clinicopathologic features, such as lymph node (LN) extent (number of stations or zones involved), LN volume (bulky, non-bulky), primary tumor size and histological subtype.

The volume and extent of N2 disease correlates with the LR rate and prognosis [30,42,54,55]. The recent meta-analysis by Liu et al. [30] showed improved OS for PORT in patients with high LN extent (multiple N2 LN metastases or multiple N2 station involvement) but not for patients with single-station N2 involvement. Lymph node ratio (LNR) (the number of pathologically positive LNs divided by the number of LNs examined) seems to be an important prognostic factor [55,56]. Furthermore, in two analyses, PORT showed a survival benefit only in pN2 patients with an LNR of 50% or more [55,56]. In a retrospective study by Wei et al. [42], PORT reduced LR and improved OS for patients in stage III NSCLC with multi-station pN2, single-station pN2 + multi-station pN1, patients with a high positive LNR > 1/3 and tumors with poor histological differentiation.

In the PORT-C trial, a preplanned exploratory analysis found a significant DFS improvement with PORT in patients with four or more LNs compared to patients with less than four LNs involved [44].

In several studies, the predictive value of absolute tumor size or pT stage in pN2 NSCLC regarding PORT was investigated, mostly with conflicting results [26,57,58,59]. The meta-analysis of Liu et al. [30] showed no significant differences in OS between the PORT and non-PORT groups for either patients with tumors >3.0 cm or those with tumors 3 cm or less.

There is also increasing evidence that there might be a benefit for PORT in persistent pN2 disease (ypN2) after induction chemotherapy (ICT). The phase 2 trial of Betticher et al. [60] found worse OS for patients with persistent N2 compared to patients with a downstaging to N0 or N1 after ICT, suggesting a possible benefit of PORT for persistent disease. Randomized trials evaluating PORT for persistent N2 disease after ICT are lacking. The role of PORT in subjects with and without pathologic complete response after ICT remains unclear [35].

The ADAURA trial found a significantly improved DFS for osimertinib in patients with EGFR-mutated completely resected stage IB to III NSCLC [16]. The benefit of PORT may be lower in patients with actionable mutations [61].

These findings indicate that treatments for stage III pN2 NSCLC should be individualized. In routine clinical practice, many criteria influence the decision-making process [62]. The aim of a recently published decision-making analysis was to identify disease characteristics in current clinical practice among European lung cancer experts for stage III pN+ NSCLC and how they impact decision making in the clinical routine regarding the use of PORT before and after the first results of the Lung ART trial presented at ESMO 2020. The most common risk factors used for decision making in the analysis were ENE and/or capsular rupture of LNs, incomplete mediastinal lymphadenectomy, multi-station LNs, high nodal tumor load and poor response to ICT [63].

The definition of complete resection has evolved over time, with findings of ENE/capsular rupture of LNs automatically being classified as incomplete resections (Rami-Porta et al. [17]). However, in clinical practice, ENE is often considered separately from otherwise completely resected tumors (otherwise R0), as has been shown among European radiation oncology experts [63]. Overall, there is ambiguity in differentiating incomplete resections from other risk factors such as ENE.

## 3. PORT after Incomplete Resection

Incomplete resection is defined by Rami-Porta et al. [17] as the presence of positive margins (R1: microscopic residual tumor, R2: macroscopic residual tumor), ENE of the tumor in the nodes removed separately or those at the margin of the main lung specimen or positive nodes left in the operative field. The definition of uncertain resection (R(uncertain)) was also created by Rami-Porta et al. [17]: no evidence of remnant tumor, but intraoperative nodal evaluation not meeting the requirements of systematic nodal dissection or lobe-specific systematic nodal dissection or involvement of the highest mediastinal node removed [17].

The justification for the presence of an ENE leading to the definition of an incomplete resection is a matter of debate. A recent analysis on the IASLC database was not able to determine the impact of ENEs on survival rates. The relevance of ENEs remains unclear, as does the interplay between ENEs and PORT. The authors concluded that analyses of a greater number of ENE cases are required to understand the prognostic impact [64].

A retrospective NCDB-based analysis of 3395 patients showed an improved OS across all nodal stages with PORT in patients with incompletely resected (R1/2) stage II-III NSCLC [65]. OS improvement was most pronounced in pN0 disease, with a 5-year OS of 41% vs. 26% with and without PORT, respectively. In another analysis of the same database with 1446 incompletely resected (R1 or R2) patients, there was only a trend in favor of sequential vs. concomitant postoperative CRT [66]. Both studies should be interpreted with caution due to the limitations and biases of registries.

A recent analysis of the NCDB registry found no significant difference in the cohort of 277 incompletely resected patients (R1: 94%, R2: 6%) between those who received chemotherapy followed by PORT alone and those who received concomitant CRT postoperatively. Most patients in this cohort had microscopic positive margins rather than gross residual disease, and it is patients in the latter group who, hypothetically, might derive the most benefit from postoperative concomitant CRT [50].

There is growing recognition that the uncertain resection status (inadequate intraoperative lymph node dissection, positivity of the highest LN, involved LNs removed in fragments, ENE) is associated with worse prognosis than R0 resection. These features might be considered as risk factors for an increased rate of local recurrence and could be potentially improved with PORT [67].

Controversial data are available for an ENE being used as a predictive marker for PORT [68]. The retrospective analysis of Moretti et al. [69] involving 83 bulky pN2 patients showed a significantly lower OS in pN2 patients with an ENE but a higher OS rate in patients without an ENE who were treated with PORT.

In the Lung ART trial, after a review of the surgical and pathological reports in accordance with IASLC, 33% of the reports did not contain any information about the ENE of the resected LNs. All patients were reclassified into R0, uncertain and R1 resection. Patients with an ENE were considered as R1 because of the insufficient description of the ENE and whether these nodes were removed separately or not. A total of 149 patients (74/250 in the PORT group, 75/243 in the control group) were reclassified as R1 (because of the ENE). The authors of the Lung ART trial are expected to report on the effect of the quality of surgery and the certainty of resection margins on the efficacy of PORT in the future.

The NCCN guidelines recommend postoperative CRT (either sequential or concurrent) for R1 resection and concurrent CRT postoperatively for R2. Depending on the stage of disease and site of R+-resection, re-excision may be considered [1,2,35,70]. The ESMO guidelines recommend PORT and adjuvant chemotherapy in patients with R1 resection. In case both chemotherapy and PORT are administered, RT may be administered before chemotherapy [11].

## 4. PORT Toxicity

Toxicity (mostly cardiopulmonary) is an important concern related to PORT, particularly as several studies could not demonstrate a clear oncologic benefit. Excessive volumes of RT, suboptimal radiation techniques, large doses and fraction sizes and non-CT-based RT planning can probably explain the excess toxicity with non-cancer-related deaths observed in previous trials with PORT.

Several retrospective databases and reviews have examined the hypothesis that more modern radiation techniques do not lead to increased deaths. They found a similar risk of intercurrent death between NSCLC patients with and without PORT [32,34,71].

An analysis of the SEER database investigated the cardiac toxicity and related mortality in 6148 patients treated with or without PORT. PORT was associated with a significantly increased number of deaths from heart disease in patients diagnosed with NSCLC between 1983 and 1988 but not in the latter cohorts [72].

The meta-analysis of the oldest trials using linear accelerators showed that the evolution from conventional 2D to 3D-RT has clearly alleviated radiation toxicity [35,37].

Subsequently, the improvements in RT technologies such as 3D conformal radiation therapy (3D-CRT), intensity-modulated radiotherapy (IMRT) or volumetric modulated arc therapy (VMAT) could have potential benefits [12,32,71,73,74].

A comparison of the dosimetric PORT plans of ten pN2 NSCLC patients using 3D-CRT, IMRT and VMAT was unable to reveal that any technique had absolute dosimetry advantages for all patients [75]. The selection of the technique should be individualized, balancing target coverage and protection of the organs at risk. The use of protons may be another step towards reducing RT-induced toxicity. Small retrospective series showed there were significantly lower RT doses to surrounding organs at risk (heart, lungs) with proton-based PORT [76,77,78,79]. In the monocentric retrospective review of Boyce-Fappiano et al. [78], the improved sparing of the heart and lung with the use of proton beam PORT was associated with improved OS. Multicenter studies randomizing patients to PORT with proton therapy versus photon therapy, including a cardiopulmonary toxicity endpoint, would be a good approach to understanding the potential benefits of proton therapy.

Unfortunately, patients in the Lung ART trial were mainly treated with 3D-CRT (89%), and only 11% of the patients in the study received IMRT, because when accrual started for the study, IMRT was not standard. IMRT has now become a standard RT technique with better dose conformity and organ at risk avoidance such as heart or lungs. In the Lung ART trial, cardiopulmonary toxicity and related death was significantly higher in the PORT group (16 patients, 16%) than in the control group (2 patients, 2%). Eleven percent of the PORT patients and 5% of the non-PORT patients had late grade 3–4 cardiopulmonary toxicities; the most common event was pneumonitis (6% in the PORT group, <1% in the control group). Further analysis will be needed to explore which patient groups might benefit most from PORT with lower toxicity [45].

## 5. Dose and Fractionation

The total RT dose, fractionation and the treated volume (including organs at risk) should also be taken into consideration in decision making.

The randomized trial by Dautzenberg et al. [30] showed a higher risk for late toxicity and intercurrent deaths using fraction sizes >2 Gy. There was a correlation between fractionation size and morbidity. The risk for non-cancer-related death was 7% in the control group (no PORT), 16–18% among patients treated with PORT with daily fractions <2 Gy and 26% among patients who had >2 Gy doses per fraction [30].

In terms of RT dose, Corso et al. [80] showed a significantly improved OS in completely resected NSCLC patients who received 45 to 54 Gy compared with patients without PORT. With RT doses of over 54 Gy, the survival was equivalent to patients treated without PORT, suggesting that these higher doses were detrimental.

## 6. Target Volume Delineation

PORT clinical target volume (CTV) must account for the lymph nodes involved according to the surgery and pathology report and should consider preoperative imaging. In cases of neoadjuvant chemotherapy, initially involved lymph node stations should be included, even in cases of downstaging.

Spoelstra et al. [81] summarized which lymph node regions should be included postoperatively for different positive lymph stations [81].

Several studies have investigated the pattern of locoregional relapse according to the tumor location. Billiet et al. [82] showed the greatest LR at LN stations 7 (18%), 4R (16%) and 10R (16%). Among left-sided tumors, LR occurred mostly bilaterally, whereas among right-sided tumors, LR was more unilateral [82].

**Figure 1:**
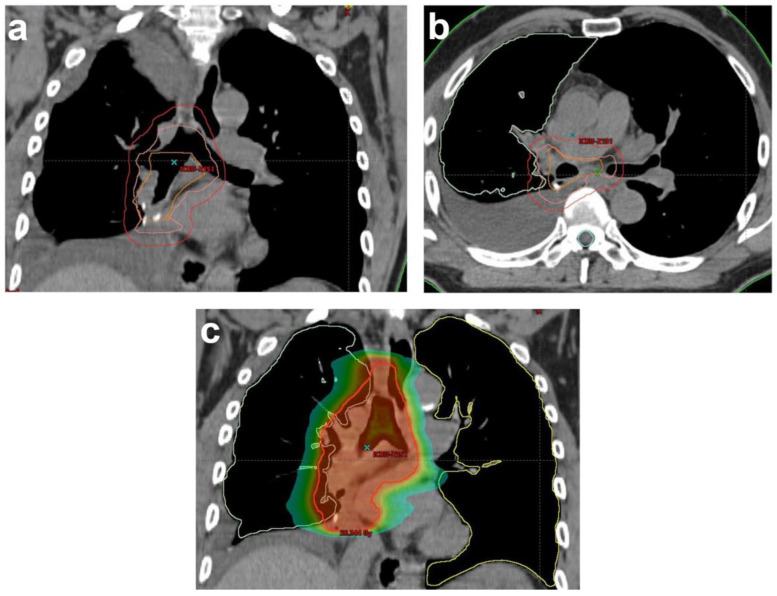
Illustration of RT planning of PORT for a completely resected NSCLC patient with histologically proven lymph nodes (2/9) in stations 7 and 10R in (**a**) coronal and (**b**) sagittal views. Delineation based on the Lung ART protocol of rCTV (orange): bronchial stump, ipsilateral hilar node region (10R) and lymph node station 7. CTV (pink): rCTV+1 cm. In this case, 4R, 7 and 10R had a maximal upper limit to the top of the aortic arc and a maximal lower limit 5 cm below the carina. PTV (red); (**c**) color wash of dose distribution ranging from 20 Gy to 57.7 Gy (prescribed dose: 54 Gy). rCTV: resected clinical tumor volume, CTV: clinical tumor volume, PTV: planning target volume.

ESTRO/ACROP guidelines suggested that CTV should include the pathologically involved and resected mediastinal lymph node stations, bronchial stump, ipsilateral hilum and ipsilateral nodal stations 4 and 7. The definition of CTV depends on the lung lobe where the tumor was located, but the bronchial stump, ipsilateral hilum, positive lymph node regions and LN stations 4 and 7 must always be included in the CTV due to their high risk of relapse [83] (Figure 1).

## 7. Conclusions

The role of PORT in patients with completely resected NSCLC with pN2 involvement remained unclear, based mostly on data from non-randomized studies and large database analyses, until the publication of the Lung ART and PORT-C trials. The older studies suggested for this patient cohort that PORT after adjuvant chemotherapy could improve overall survival providing that a conformal RT technique such as 3D or IMRT would be related with less cardiopulmonary toxicity.

The Lung ART and PORT-C trials represent robust evidence that 3D conformal PORT should not be generally recommended in patients with resected stage III pN2 NSCLC patients. Data indicate a significantly lower locoregional relapse rate with PORT without translating into a survival benefit. It is a matter of ongoing debate as to whether PORT might be beneficial for selected patients with high risk features, such as more than or equal to four LN metastases, multiple-station N2 or persistent N2 disease after ICT [61,67]. It is unclear whether PORT could improve the outcome for patients with ENE. The optimal RT technique for PORT, such as IMRT or intensity-modulated proton therapy, and the role for PORT in selected high-risk patients should be evaluated. Future research should focus on defining the profile of optimal candidates who might benefit from PORT.

## Figures and Tables

**Table 1:** Studies evaluating PORT in stage I–III pN0–pN2 NSCLC patients.

Trial	Year of Publication	Disease Stage	Study Design	Patient Number	Inclusion Period	Resection Status	Chemotherapy	Technique PORT	Dose (Gy)	OS	PORT	Non-PORT
Van Houtte	1980	pN0–pN2	RCT	224	NA	NA	No	Cobalt	60	5 Y (n.s.)	24%	43%
LCSG 773	1986	pN1–pN2	RCT	230	NA	NA	No	Cobalt, Linac	50	n.s.	NA	NA
Debevec	1996	pN2	RCT	74	1988–1992	R0	No	Linac	30	n.s.	NA	NA
Lafitte	1996	pN0	RCT	163	1985–1991	NA	No	Cobalt, Linac	45–60	5 Y (n.s.)	NA	NA
Stephens	1996	pN1–pN2	RCT	308	1986–1993	NA	No	Cobalt, Linac	40	n.s.	NA	NA
Dautzenberg	1999	pN0–pN2	RCT	728	NA	NA	No	Cobalt, Linac	60	5 Y (s.)	30%	43%
Feng	2000	pN1–pN2	RCT	366	1982–1995	NA	No	Cobalt, Linac	60	5 Y (n.s.)	43%	41%
Trodella	2002	pN0	RCT	104	1989–1997	NA	No	Linac	50	5 Y (n.s.)	67%	58%
Lally	2006	pN2	R	1987	1988–2002	R0–1–2	NA	Linac	NA	5 Y (s.)	27%	20%
Mayer	2006	pN2	RCT	155	NA	R0	No	Linac, 3D	50–56	2 Y (n.s.)	46%	41%
Perry	2007	pN2	RCT	37	1998–2000	R0	adj (100%)	NA	50	1 Y (n.s.)	74%	72%
Douillard (ANITA)	2008	pN2	post hoc R	224	NA	R0	adj (48.4%)	Linac	45–60	5 Y	47%	34%
Shen	2013	pN2	RCT	135	2004–2009	R0	adj	NA	50	5 Y (n.s.)	38%	28%
Corso	2014	pN2	R	6979	1998–2006	R0	neo and or adj (34%)	Linac (3D or IMRT)	54 (median)	5 Y (s.)	34%	28%
Robinson	2015	pN2	R, multicenter	4483	2006–2010	R0	adj (100%)	Linac, 3D	45–83	5 Y (s.)	39%	35%
Feng	2015	pN2	R	357	2005–2012	R0	adj	Linac, 3D	50 (median)	5 Y (s.)	57%	35%
Billiet	2016	pN2	R	150	1998–2012	R0–1–2	neo (100%)	Linac, 3D	50–66	5 Y (n.s.)	32%	42%
Park	2016	pN2	R	240	2006–2012	R0–1	adj	Linac, 3D	50 (median)	5 Y (n.s.)	NA	NA
Herskovic	2017	pN2	R, multicenter	2691	2004–2013	R0	adj	NA	50 (median)	median OS	53 mo	45 mo
Sun	2017	pN2	RCT	101	2009–2014		adj	NA	50	n.s.	74 mo	84 mo
Wei	2020	pN2	R, single center	183	2013–2016	R0	adj	Linac, IMRT	50 (median)	2 Y (s.)	78%	62%
Wang	2021	pN2	R	142	2014–2015	R0	adj	Linac, 3D	45–54	5 Y (s.)	32%	27%
Van Zandwijk EORTC	not published, only abstract	pN1-pN2	RCT	106	NA	NA	NA	Linac	56	NA	NA	NA
Le Pechoux Lung ART	2021	pN2	RCT	501	2007–2018	R0	adj (96%)	Linac, 3D (89%), IMRT (11%)	54	3 Y (n.s.)	67%	69%
Hui PORT-C	2021	pN2	RCT	364	2009–2017	R0	adj (100%)	Linac, 3D (11%) or IMRT (89%)	50	3 Y (n.s.)	78%	83%

NA: information not available, RCT: randomized controlled trial, R: retrospective study, neo: neoadjuvant chemotherapy. adj: adjuvant chemotherapy, linac: linear accelerator, IMRT: intensity-modulated radiation therapy, OS: overall survival, Y: year, s.: significant, n.s.: not significant, mo: months.
